# Chitosan as a Renewable Heterogeneous Catalyst for the Knoevenagel Reaction in Ionic Liquid as Green Solvent

**DOI:** 10.5402/2012/928484

**Published:** 2012-07-17

**Authors:** Nam T. S. Phan, Ky K. A. Le, Thien V. Nguyen, Nhan T. H. Le

**Affiliations:** Department of Chemical Engineering, Ho Chi Minh City University of Technology, VNU-HCM, 268 Ly Thuong Kiet, District 10, Ho Chi Minh City 70350, Vietnam

## Abstract

The combination of chitosan as a renewable heterogeneous catalyst and ionic liquid as a “green” solvent was employed for the Knoevenagel reaction. The chitosan catalyst was characterized by various techniques, including X-ray powder diffraction (XRD), scanning electron microscopy (SEM), transmission electron microscopy (TEM), thermogravimetric analysis (TGA), Fourier transform infrared spectroscopy (FT-IR), and elemental analysis. Excellent conversions were achieved under mild conditions without the need for an inert atmosphere. There was no contribution from leached active species, and conversion was only being possible in the presence of the solid catalyst. The chitosan catalyst as well as the ionic liquid solvent could be recovered in essentially pure form after being used in the reaction, and each of them could be reused several times without a significant degradation in efficiency.

## 1. Introduction

Room temperature ionic liquids have been considered as potential green alternatives to conventional volatile organic solvents during the last decade [1–5]. They exhibit several advantages such as negligible vapor pressure, excellent ability to dissolve organic compounds, ease of separation from products, and potential for recycling [6–8]. A variety of ionic liquids have been investigated, generally consisting of salts of organic cations, for example, tetraalkylammonium, alkylpyridinium, 1,3-dialkylimidazolium, tetraalkylphosphonium [2, 9]. During the past few years, several organic transformations have been carried out using ionic liquids as environmentally benign solvents, such as hydrogenation [10], oxidation [11–14], Heck cross-coupling reaction [15, 16], Suzuki reaction [17], Sonogashira reaction [18], Diels-Alder reaction [19], aldol condensation [20], alkylation [21–23], Micheal addition [24], oxa-Michael addition [25], Schmidt reaction [26], ring-closing metathesis [27], esterification reaction [28, 29], and enzyme-catalyzed organic reactions [30–33]. However, since the application of the first ionic liquid sample as solvent for organic transformations, research works have been mostly focused on homogeneous catalysis in ionic liquids. Indeed, reports on organic reactions using heterogeneous catalysts in ionic liquids as solvents have been very limited in the literature [34–38].

The Knoevenagel condensation between aldehydes or ketones with activated methylene compounds is one of important carbon-carbon forming reactions in organic synthesis [39, 40]. Conventionally, this reaction is catalyzed by alkali metal hydroxides or by organic bases under homogeneous conditions with the attendant difficulties in catalyst recovery and recycling [41]. Over the last few years, several solid catalysts have been employed for this reaction such as zeolites exchanged with alkylammonium cations [42], amine-functionalized mesoporous zirconia [43], mesoporous titanosilicate [44], basic MCM-41 silica [45–47], acid-base bifunctional mesoporous MCM-41 silica [48], nanocrytalline ceria-zirconia [49], amine-functionalized superparamagnetic nanoparticles [50], organic-inorganic hybrid silica materials [51], and metal-organic frameworks [52, 53]. Chitosan, a biomaterial derived from crustacean shells, offers the advantages of being renewable and biodegradable, as well as being relatively cheap and of low toxicity [54]. It was used as a “green” catalyst support for several transitional metal-catalyzed reactions [55–58], and it was also used as a solid base catalyst for the aldol condensation reaction [59]. However, all of these processes were still carried out in conventional volatile organic solvents. In this paper, we wish to report the combination of chitosan as a renewable heterogeneous catalyst and ionic liquid as a green solvent for the Knoevenagel reaction. The chitosan catalyst as well as the ionic liquid solvent could be recovered in essentially pure form after being used in the reaction. Both the catalyst and the solvent could be reused several times without a significant degradation in performance.

## 2. Experimental

### 2.1. Materials and Instrumentation

All reagents and starting materials were obtained commercially from Sigma-Aldrich and Merck, and they were used as received without any further purification unless, otherwise, noted. Chitosan was kindly donated by MKVN Chemicals Company (authorized distributor of COGNIS-HENKEL, Germany). Nitrogen physisorption measurements were conducted using a Quantachrome 2200e system. Samples were pretreated by heating under vacuum at 150°C for 3 h. A Netzsch Thermoanalyzer STA 409 was used for thermogravimetric analysis (TGA) with a heating rate of 10°C/min from 30 to 900°C in air. X-ray powder diffraction (XRD) patterns were recorded using a Cu K*α* radiation source on a D8 Advance Bruker powder diffractometer. Scanning electron microscopy studies were conducted on a JSM 740 Scanning Electron Microscope (SEM). Transmission electron microscopy studies were performed using a JEOL JEM 1400 Transmission Electron Microscope (TEM) at 100 kV. The chitosan samples were dispersed on holey carbon grids for TEM observation. Fourier transform infrared (FT-IR) spectra were obtained on a Bruker TENSOR37 instrument, with samples being dispersed on potassium bromide pallets. ^1^H and ^13^C NMR spectra were recorded using a Bruker AV 500 spectrometer. MS spectra were performed on a Thermo Finigan TSQ7000 triple quadrupole.

Gas chromatographic (GC) analyses were performed using a Shimadzu GC 17-A equipped with a flame ionization detector (FID) and an DB-5 column (length = 30 m, inner diameter = 0.25 mm, and film thickness = 0.25 *μ*m). The temperature program for GC analysis heated samples from 60 to 200°C at 20°C/min and held them at 150°C for 1 min; then heated them from 150 to 160°C at 1°C/min and held them at 200°C for 2 min; then heated them from 200 to 300°C at 50°C/min and held them at 300°C for 4 min. Inlet and detector temperatures were set constant at 300°C. *p*-Xylene was used as an internal standard to calculate reaction conversions. GC-MS analyses were performed using a Hewlett Packard GC-MS 5972 with an RTX-5MS column (length = 30 m, inner diameter = 0.25 mm, and film thickness = 0.5 *μ*m). The temperature program for GC-MS analysis heated samples from 60 to 280°C at 10°C/min and held them at 280°C for 2 min. Inlet temperature was set constant at 280°C. MS spectra were compared with the spectra gathered in the NIST library.

### 2.2. Synthesis of Ionic Liquid

1-Butyl-3-methylimidazolium bromide ([BMIM][Br]) was prepared from the reaction of *N*-methylimidazole and *n*-butyl bromide, according to a literature procedure [60, 61]. A plastic conical flask containing a mixture of [BMIM][Br] (25.10 g, 0.115 mol) and distilled water (50 mL) was immersed in an ice bath for 30 min. Hexafluorophosphoric acid (HPF_6_) 60% (20 mL, 0.147 mol) and water (50 mL) were then added dropwise to prevent the temperature from rising significantly. After stirring for 12 h at room temperature, the upper acidic aqueous layer was separated by decantation and the lower ionic liquid phase was washed with cold water (10 × 50 mL) until the washings were no longer acidic [61]. The ionic liquid was then heated under vacuum at 60°C to remove any excess water, affording 27.32 g of 1-butyl-3-methylimidazolium hexafluorophosphate ([BMIM][PF_6_]) (83% yield).

^1^H NMR (500 MHz, DMSO-d6): *δ* = 0.91 (t, 3H; CH_3_), 1.26 (m, 2H; CH_2_CH_3_), 1.77 (m, 2H; CH_2_CH_2_CH_3_), 3.85 (s, 3H; N–CH_3_), 4.16 (m, 2H; N–CH_2_), 7.67 (t, 1H; N–CH=C), 7.73 (t, 1H; N–CH=C), 9.07 (s, 1H, N–CH=N). ^13^C NMR (125 MHz, DMSO-d6): *δ* = 13.14 (C–CH_3_), 18.71 (CH_2_), 31.27 (CH_2_), 35.65 (N–CH_3_), 48.51 (N–CH_2_), 122.19 (C=C–N), 123.54 (C=C–N), 136.44 (N–C=N). MS (ESI): *m/z* 139 [BMIM]^+^, 423 [(BMIM)_2_PF_6_]^+^.

Using a similar procedure, 1-hexyl-3-methylimidazolium hexafluorophosphate ([HMIM][PF_6_]), and 1-octyl-3-methylimidazolium hexafluorophosphate ([OMIM][PF_6_]) ionic liquids were synthesized in a yield of 83% and 85%, respectively.

^1^H NMR (500 MHz, DMSO-d6) for [HMIM][PF_6_]: *δ* = 0.87 (t, 3H; CH_3_), 1.27 (m, 6H; CH_2_CH_2_CH_2_), 1.78 (m, 2H; CH_2_), 3.85 (s, 3H; N–CH_3_), 4.15 (m, 2H; N–CH_2_), 7.77 (t, 1H; N–CH=C), 7.73 (t, 1H; N–CH=C), 9.07 (s, 1H, N–CH=N). ^13^C NMR (125 MHz, DMSO-d6): *δ* = 13.71 (C–CH_3_), 21.80 (CH_2_), 25.09 (CH_2_), 29.27 (CH_2_), 30.49 (CH_2_), 35.65 (N–CH_3_), 48.79 (N–CH_2_), 122.19 (C=C–N), 123.54 (C=C–N), 136.43 (N–C=N). MS (ESI): *m/z* (%) 167 [HMIM]^+^, 479 [(HMIM)_2_PF_6_]^+^.

^1^H NMR (500 MHz, DMSO-d6) for [OMIM][PF_6_]: *δ* = 0.86 (t, 3H; CH_3_), 1.27 (m, 10H; CH_2_CH_2_CH_2_CH_2_CH_2_), 1.78 (m, 2H; CH_2_), 3.85 (s, 3H; N–CH_3_), 4.15 (m, 2H; N–CH_2_), 7.67 (t, 1H; N–CH=C), 7.74 (t, 1H; N–CH=C), 9.08 (s, 1H, N–CH=N). ^13^C NMR (125 MHz, DMSO-d6): *δ* = 13.87 (C–CH_3_), 22.39 (CH_2_), 26.09 (CH_2_), 28.77 (CH_2_), 28.84 (CH_2_), 30.14 (CH_2_), 31.50 (CH_2_), 36.64 (N–CH_3_), 50.00 (N–CH_2_), 121.86 (C=C–N), 123.64 (C=C–N), 137.08 (N–C=N). MS (ESI): *m/z* 195 [OMIM]^+^, 535 [(OMIM)_2_PF_6_].

### 2.3. Catalytic Studies

The Knoevenagel reaction between benzaldehyde and malononitrile using the chitosan catalyst was carried out in a magnetically stirred round bottom flask. Unless, otherwise, stated, a mixture of chitosan (91 mg g, 20 mol%), benzaldehyde (0.2 mL, 1.9 mmol), and *p*-xylene (0.2 mL) as an internal standard was placed into a 25 mL flask containing 3 mL [BMIM][PF_6_]. The catalyst concentration was calculated with respect to the amino/benzaldehyde molar ratio. The reaction vessel was stirred for 30 min to disperse the chitosan catalyst in the liquid phase. Malononitrile (0.5 mL, 7.6 mmol) was then added, and the resulting mixture was stirred at room temperature for 6 h. Reaction conversion was monitored by withdrawing aliquots from the reaction mixture at different time intervals, quenching with acetone, filtering through a short silica gel pad, analyzing by GC with reference to *p*-xylene, and further confirming product identity by GC-MS. The reaction mixture was washed with diethyl ether (5 × 15 mL). The chitosan catalyst was then separated by simple centrifugation, washed with copious amounts of anhydrous ethanol, dried at 60°C overnight, and reused if necessary. For the leaching test, a catalytic reaction was stopped after 0.5 h, analyzed by GC, and centrifuged to remove the solid catalyst. The reaction solution was then stirred for further 5.5 h at room temperature. Reaction progress, if any, was monitored by GC as previously described.

## 3. Results and Discussion

### 3.1. Catalyst Characterization

**Figure 1:**
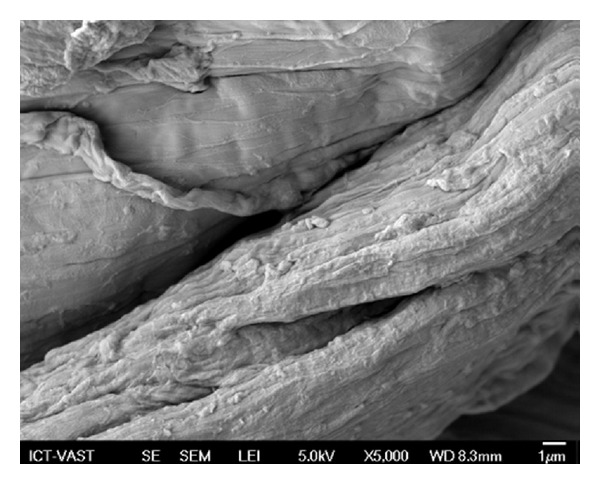
SEM micrograph of the chitosan catalyst.

**Figure 2:**
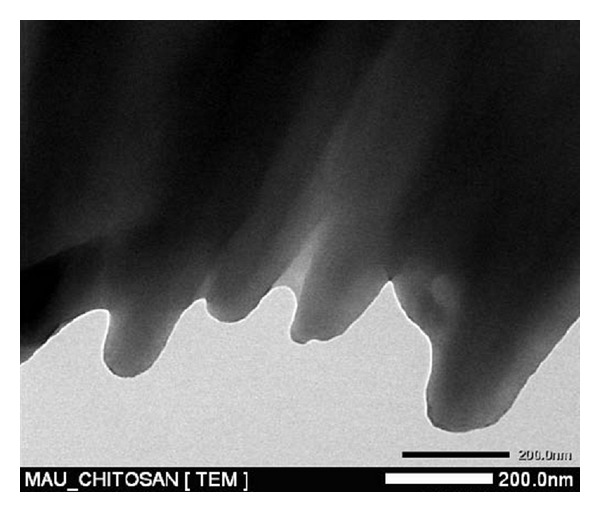
TEM micrograph of the chitosan catalyst.

**Figure 3:**
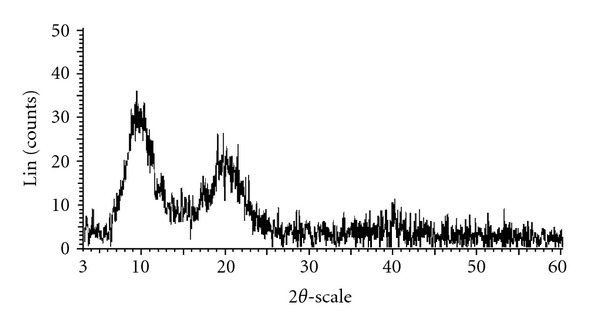
X-ray powder diffractogram of the chitosan catalyst.

**Figure 4:**
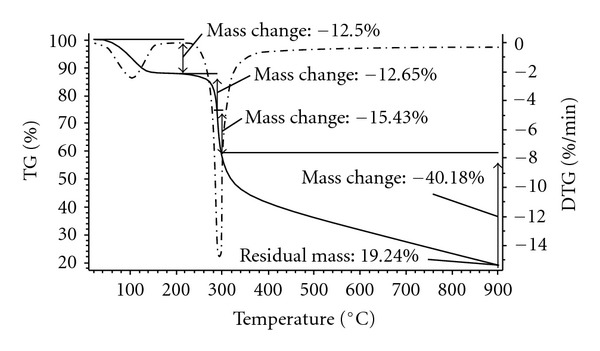
TGA analysis of the chitosan catalyst.

The chitosan catalyst was characterized using a variety of different techniques, including SEM, TEM, XRD, FT-IR, TGA, nitrogen physisorption measurements, and elemental analysis. The morphology of the chitosan was observed from the SEM micrograph, exhibiting an uneven surface with straps and shrinkage (Figure 1). As expected, the TEM micrograph revealed that the chitosan catalyst possessed a nonporous structure (Figure 2). No measurable mesoporosity was observed on the nitrogen physisorption measurements. Indeed, several approaches have been developed to achieve a porous structure for chotosan-based materials [62–66]. As inspired by green chemistry principles, unmodified chitosan was used in this research. The crystallinity of the chitosan catalyst was analyzed by XRD. Two characteristic peaks [67] at 2*θ* = 10.5° and 2*θ* = 20.5° were observed on the XRD diffractogram of the catalyst (Figure 3). These two broad diffraction peaks are normally considered as typical fingerprints of semicrystalline chitosan [68]. TGA result indicated that the chitosan catalyst degraded in two stages during the heating process from 30 to 900°C in air. The first weight loss of 12.5% was due to the residual and physisorbed water. The second step started at around 260°C, with a decomposition peak temperature of around 300°C and a total weight loss of 68% (Figure 4). The thermal stability of the chitosan catalyst was, therefore, in good agreement with the literature [69], ensuring that the catalyst could be used across a wide temperature range for liquid-phase reactions.

**Figure 5:**
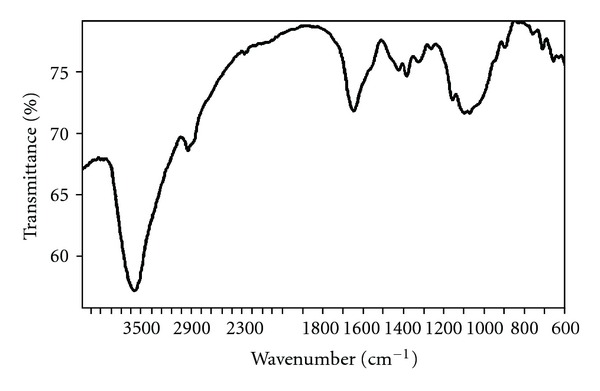
FT-IR spectra of the chitosan catalyst.

As expected, the FT-IR spectra of the catalyst indicated characteristic bands of chitosan. Broad peaks near 3450 cm^−1^ were attributed to the O–H stretching vibration of the hydroxyl groups, as well as the inter- and extramolecular hydrogen bonding of chitosan molecules [70]. These broad bands were also indicative of the presence of physisorbed water in the material. There would also exist the contribution of the –NH_2_ group for the band in the region of 3000–3400 cm^−1^, which was overlapped by the O–H stretching vibration. The weak band near 2922 cm^−1^ was attributed to the C–H stretching vibration. The band near 1646 cm^−1^ was assigned to the vibration of amide one groups (Figure 5). Elemental analysis of the catalyst indicated a total nitrogen loading of 5.2 mmol/g. The degree of N-deacetylation of the chitosan (i.e., the average number of D-glucosamine units per 100 monomers, expressed as a percentage) was found to be 87%, as estimated following Block's method [71]. The amino group loading in the chitosan catalyst was therefore calculated to be 4.5 mmol/g. For the reason of simplicity, the amino group loading was used as an elemental tag for the catalyst. However, it should be noted that not all of these amino groups were accessible to the reactants during the course of the reaction.

### 3.2. Catalytic Studies

**Figure 6:**
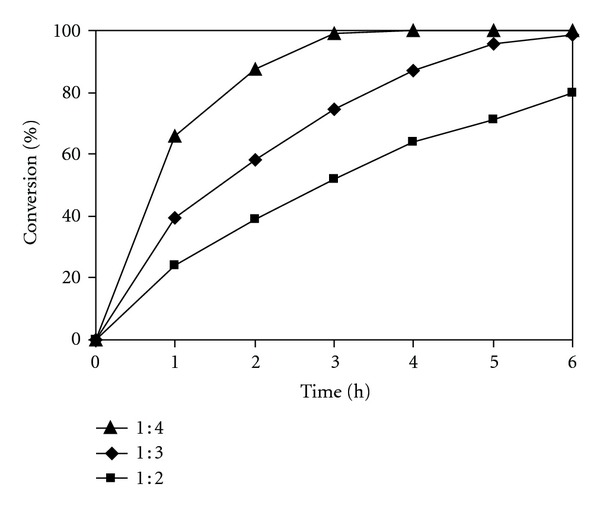
Effect of benzaldehyde: malononitrile molar ratio on reaction conversion.

**Scheme 1:**
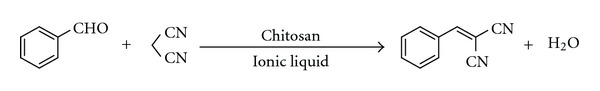
Knoevenagel reaction of benzaldehyde with malononitrile using the chitosan catalyst.

The chitosan catalyst was assessed for its activity in the Knoevenagel condensation between benzaldehyde and malononitrile to form benzylidene malononitrile as the principal product (Scheme 1). As inspired by green chemistry principles, it was decided to carry out the reaction in the [BMIM][PF_6_] ionic liquid as solvent at room temperature. The ionic liquid solvent was synthesized and characterized according to a literature procedure [60, 61]. Aliquots were withdrawn from the reaction mixture at different time intervals and analyzed by GC, showing the kinetic data during the course of the reaction. When investigating the chitosan-catalyzed Knoevenagel reaction in ionic liquid, there would be several factors that should be taken into consideration. Initial studies addressed the effect of the benzaldehyde : malononitrile molar ratio on reaction conversions, having carried out the reaction at 20 mol% chitosan catalyst with molar ratios of 1 : 2, 1 : 3, and 1 : 4, respectively. It was found that the Knoevenagel reaction using the reagent molar ratio of 1 : 3 could afford a conversion of 98% after 6 h. More than 99% conversion was achieved after 3 h at the reagent ratio of 1 : 4. As expected, decreasing the reagent molar ratio to 1 : 2 resulted in a drop in reaction rate, though 80% conversion was still observed after 6 h (Figure 6).

**Figure 7:**
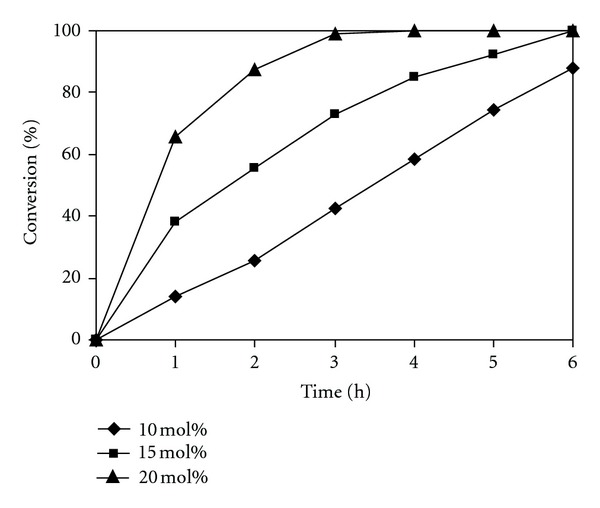
Effect of catalyst concentration on reaction conversion.

With this result in mind, it was then decided to investigate the effect of catalyst concentration on the reaction conversion, having carried out the reaction at 10 mol%, 15 mol%, and 20 mol% catalyst, respectively, at the reagent molar ratio of 1 : 4. As mentioned earlier, the chitosan catalyst had a nonporous structure, and, therefore, not all of amino groups in the polymer chain were accessible to the reactants during the course of the reaction. Indeed, further investigations would be needed to quantify the real active amino groups in the Knoevenagel reaction carried out in ionic liquid. However, for the reason of simplicity, the amino group concentration calculated from the total nitrogen loading and the degree of N-deacetylation was still used as an elemental tag for the chitosan catalyst. It was observed that quantitative conversion of benzaldehyde was achieved after 6 h for the reaction using 15 mol% catalyst. As expected, it was observed that increasing the catalyst concentration to 20 mol% led to a significant reaction rate enhancement, with more than 99% conversion being obtained after 3 h. The Knoevenagel reaction at 10 mol% catalyst concentration occurred with slower rate, though 88% conversion was still afforded after 6 h (Figure 7). Formentin and co-workers previously employed more than one equivalent of KOH catalyst for the homogeneous Knoevenagel reaction between benzaldehyde and malononitrile carried out in ionic liquid [72]. Tojo and coworkers previously employed 20 mol% glycine as a homogeneous catalyst for the Knoevenagel reaction in ionic liquid [73]. Indeed, it was previously reported that concentration of base catalysts used for the Knoevenagel reaction could be in the range from less than 1 mol% to 40 mol%, depending on the nature of the catalyst [43, 44, 74, 75]. The catalyst concentration used in this study was, therefore, in good agreement with the literature.

**Figure 8:**
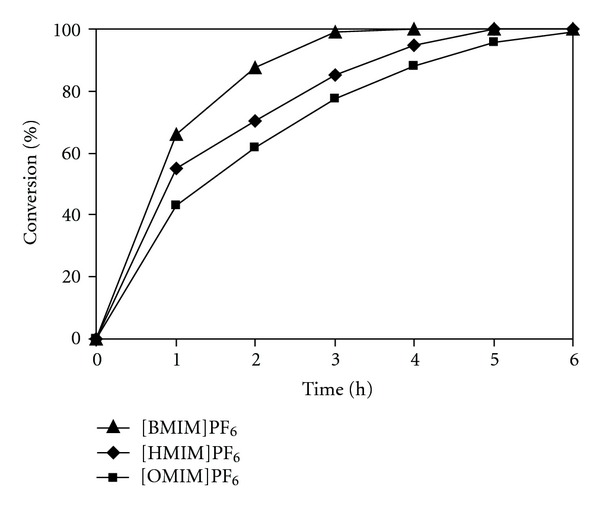
Effect of solvent on reaction conversion.

The effect of different solvents on organic transformations using heterogeneous catalysts could normally be crucial, depending on the nature of the catalyst material [50, 76]. Macquarrie and Jakson previously reported that the Knoevenagel reaction using silica-based catalysts could only proceed in a limited range of solvents, and the best reaction rate was observed in nonpolar solvents [77]. In contrast, Corma and coworkers previously showed that the reaction rate of the Knoevenagel reaction using solid catalysts decreased significantly in nonpolar solvents, and higher conversions were achieved in more polar solvents [78–80]. Gascon and coworkers reported similar effect, where polar solvents favored the reaction rate of the heterogeneous Knoevenagel condensation, and the reaction occurred with difficulty in nonpolar solvents [52, 81]. Dong and coworkers also demonstrated that the Knoevenagel reaction using a polymer-based catalyst proceeded readily in ethanol [82]. It was, therefore, decided to investigate the effect of different ionic liquid solvents on the Knoevenagel reaction using the chitosan catalyst. The reaction was carried out using 20 mol% catalyst in there ionic liquids, including [BMIM][PF_6_], [HMIM][PF_6_], and [OMIM][PF_6_], respectively. It was found that the reaction rate decreased with the solvent order: [BMIM][PF_6_] > [HMIM][PF_6_] > [OMIM][PF_6_]. The reaction carried out in [HMIM][PF_6_] afforded more than 99% conversion after 5 h, while 99% conversion was observed for the reaction in [OMIM][PF_6_] (Figure 8).

**Figure 9:**
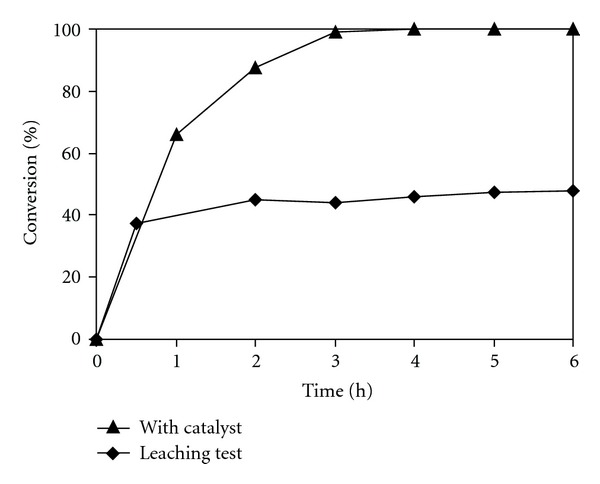
Leaching test indicated no contribution from homogeneous catalysis of active species leaching into reaction solution.

For a liquid-phase organic transformation using a solid catalyst, an important problem that should be taken into account is the possibility that some of active sites could dissolve into the solution during the course of the reaction. In fact, these leached species might contribute significantly to the catalytic reaction, and the reaction might not be truly heterogeneous with respect to the substrates and products. [50]. It should be noted that several amines previously exhibited high activity in the Knoevenagel reaction [83]. However, it was apparent that these homogeneous catalysts could not be recycled and reused for the reaction, and, therefore, they were practically undesirable. In order to determine if the Knoevenagel reaction using the chitosan catalyst in ionic liquid was truly heterogeneous, an experiment was performed using a simple centrifugation during the course of the reaction. After the chitosan catalyst was separated from the reaction mixture, if the catalytic reaction continued, this would indicate that the active species were from the solution rather than from the solid catalyst. The organic phase was separated from the solid catalyst after 0.5 h reaction time by simple centrifugation, having used 20 mol% of fresh chitosan catalyst. The reaction solution was then transferred to a new reactor vessel, stirred for an additional 5.5 h at room temperature with aliquots being sampled at different time intervals, and analyzed by GC. It was found that, within experimental errors, no further reaction was observed after the solid chitosan catalyst was removed from the reaction mixture. The leaching test result indicated that the Knoevenagel reaction carried out in ionic liquid could only proceed in the presence of the solid chitosan catalyst, and there was no contribution from active species dissolved in the reaction solution during the course of the reaction (Figure 9).

**Figure 10:**
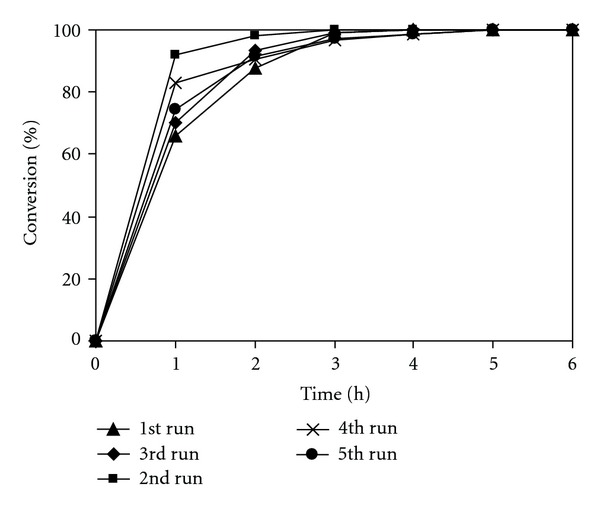
Recycling of both chitosan catalyst and ionic liquid solvent.

When using solid catalysts for organic transformations, issues that should be considered are the ease of separation as well as the deactivation and reusability of the catalyst. In fact, active lifetimes should be an important characteristic for both heterogeneous and homogeneous catalysts. Ionic liquids have been considered as green solvents not only due to their nonvolatile nature, minimizing emission of toxic organic compounds, but also because of their recyclability. The chitosan catalyst and the ionic liquid solvent were, therefore, investigated for recoverability and reusability in the Knoevenagel reaction over five successive runs. The Knoevenagel reaction was carried out using 20 mol% chitosan catalyst in the [BMIM][PF_6_] ionic liquid at room temperature. Aliquots were withdrawn from the reaction mixture at different time intervals and analyzed by GC to observe the kinetic data during the course of the reaction. After the reaction, product and unreacted starting materials were separated from the reaction mixture by extraction with diethyl ether. The mixture of chitosan catalyst and ionic liquid solvent was then washed several times with diethyl ether and reused in further reaction under identical conditions to those of the first run. Interestingly, it was found that the catalyst and solvent system could be reused without significant degradation in catalytic activity. Quantitative reaction conversion was still achieved at the fifth run using the recovered catalyst and solvent system (Figure 10).

**Figure 11:**
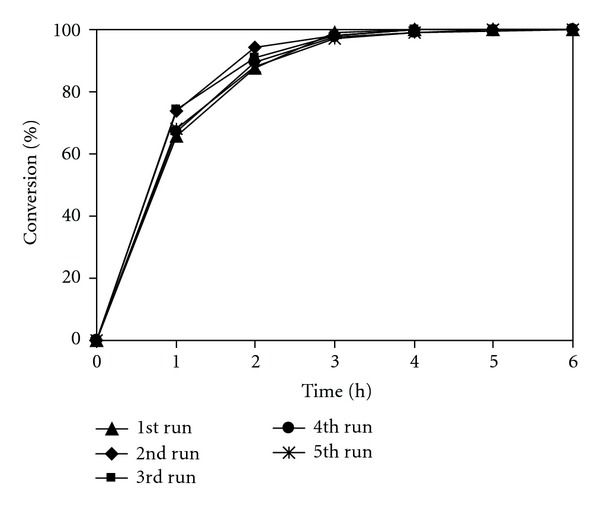
Recycling of the chitosan catalyst with fresh ionic liquid solvent for each run.

**Figure 12:**
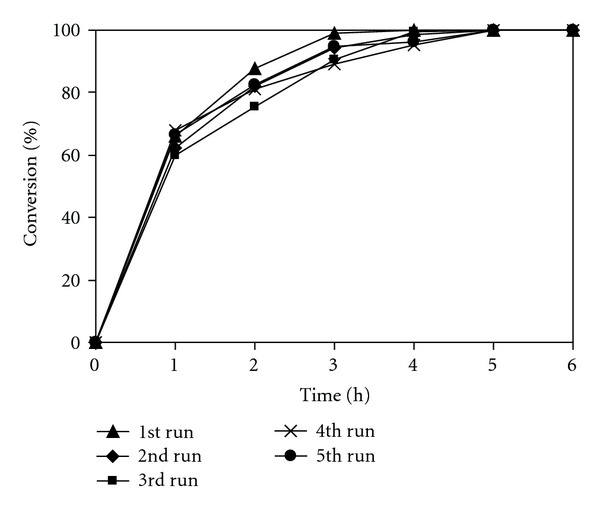
Recycling of the ionic liquid solvent with fresh chitosan catalyst for each run.

As mentioned earlier, research works have been mostly focused on homogeneous catalysis in ionic liquids, and the number of reports covering the heterogeneous catalysis in ionic liquids are limited in the literature. Santamarta and coworkers previously carried out the Knoevenagel reaction using 20 mol% glycine as a homogeneous catalyst in ionic liquid [73]. The mixture of glycine and ionic liquid could be recycled and reused several times. Formentin and coworkers also reported the recyclability of the mixture of KOH and ionic liquid in the Knoevenagel reaction [72]. Indeed, it was previously reported that the mixture of homogeneous catalysts and ionic liquid solvents could be recycled and reused in several organic transformations [3, 6–8]. However, it should be emphasized that the homogeneous catalyst could not be separated from the mixture, and hence it could not be recovered in essentially pure form after being used in the reaction. We, therefore, decided to investigate the recovery of the chitosan catalyst from the reaction mixture, and its reusability in the ionic liquid-mediated Knoevenagel reaction. After the reaction, the catalyst was separated by simple centrifugation, then washed with copious amounts of anhydrous ethanol to remove any physisorbed reagents, and dried at 60°C overnight. The recovered chitosan catalyst was reused in further reaction using fresh ionic liquid solvent for each run under identical conditions to those of the first run. It was found that the catalyst could be reused without significant degradation in activity (Figure 11). The recovery and recyclability of the ionic liquid were also investigated. After each run, the catalyst was separated by simple centrifugation, and the ionic liquid was washed several times with diethyl ether to remove the product and unreacted starting materials. The recovered ionic liquid was then reused in further reaction using fresh chitosan catalyst for each run under identical conditions to those of the first run. It was also observed that the ionic liquid could be reused without loss of efficiency (Figure 12).

## 4. Conclusions

In conclusion, we have demonstrated the application of chitosan as a renewable solid catalyst for the Knoevenagel condensation between benzaldehyde with malononitrile to form benzylidene malononitrile as the principal product. The chitosan catalyst was characterized using a variety of different techniques, including FT-IR, TEM, SEM, XRD, TGA, and elemental analysis. The reaction was carried out in ionic liquid as a “green” solvent. Excellent conversions were achieved under mild conditions without the need for an inert atmosphere. The reaction could only proceed in the presence of the solid chitosan catalyst, and there was no contribution from leached active species in the reaction solution. To the best of our knowledge, the combination of chitosan as a renewable heterogeneous catalyst and ionic liquid as a “green” solvent for the Knoevenagel reaction was not previously mentioned in the literature. The chitosan catalyst as well as the ionic liquid solvent could be recovered in essentially pure form after being used in the reaction, and they could be reused several times without a significant degradation in efficiency. Current research in our laboratory has focused on the application of several heterogeneous catalysts for a wide range of organic transformations in ionic liquids.
